# The child’s experience of becoming ill with COVID-19*

**DOI:** 10.1590/1980-220X-REEUSP-2023-0165en

**Published:** 2024-02-02

**Authors:** Juliana Barony da Silva, Nayara Luiza Henriques, Felipe Leonardo Rigo, Gonçalo Miguel Cordeiro Duarte Guerreiro, Sérgio Joaquim Deodato Fernandes, Elysangela Dittz Duarte

**Affiliations:** 1 Universidade Federal de Minas Gerais, Escola de Enfermagem, Departamento de Enfermagem Materno-infantil e Saúde Pública, Belo Horizonte, MG, Brazil.; 2 Universidade Católica Portuguesa, Faculdade de Ciências da Saúde e Enfermagem, Centro de Investigação Interdisciplinar em Saúde, Lisboa, Portugal.

**Keywords:** Child, COVID-19, Coronavirus, Nursing, Qualitative Research, Niño, COVID-19, Coronavirus, Enfermería, Investigación Cualitativa, Criança, COVID-19, Coronavirus, Enfermagem, Pesquisa Qualitativa

## Abstract

**Objective:**

To understand the experience of children when they become ill with COVID-19.

**Method:**

Qualitative-descriptive and exploratory study, guided by the World Health Organization’s concept of Quality of Life. Twenty-four children participated, aged between seven and nine years, 11 months and 29 days, diagnosed with COVID-19. Data were collected through semi-structured interviews and underwent deductive thematic analysis.

**Results:**

The children understood what COVID-19 is, its high lethality and transmissibility, and its forms of contagion and prevention. COVID-19 has been identified as something negative. The experience of children becoming ill with COVID-19 was permeated by changes in their routines, especially due to having activities limited to the home, emotional changes, and physical symptoms due to infection with the virus.

**Conclusion:**

The children understood the seriousness of the pandemic and identified the changes that had occurred. They also expressed understanding about the disease and its control. Knowing children’s experience of illness can guide care actions aimed at them, recognizing that children’s adequate understanding of what they experience can contribute to coping with illness and their participation in control actions.

## RESUMO

**Objetivo:**

Conhecer a experiência de crianças em seu adoecimento por COVID-19.

**Método:**

Estudo qualitativo descritivo e exploratório, guiado pelo conceito de Qualidade de Vida da Organização Mundial de Saúde. Participaram 24 crianças, com idades entre sete e nove anos, 11 meses e 29 dias, diagnosticadas com COVID-19. Os dados foram coletados mediante entrevista semiestruturada e submetidos à análise temática dedutiva.

**Resultados:**

As crianças compreendiam o que é a COVID-19, a sua alta letalidade e transmissibilidade e ainda suas formas de contágio e prevenção. A COVID-19 foi identificada como algo negativo. A experiência de adoecimento de crianças por COVID-19 foi permeada por mudanças em suas rotinas, sobretudo por ter as atividades limitadas ao domicílio, alterações emocionais e sintomas físicos devido à infecção pelo vírus.

**Conclusão:**

As crianças compreenderam a gravidade da pandemia e identificaram as mudanças ocorridas. Também manifestaram entendimento sobre a doença e o seu controle. Conhecer a experiência de adoecimento de crianças pode orientar ações de cuidado direcionadas a elas, reconhecendo que entendimento adequado da criança sobre o que ela vivencia pode contribuir para o enfrentamento do adoecimento e para sua participação nas ações de controle.

## RESUMEN

**Objetivo:**

Conocer la experiencia de los niños cuando enferman de COVID-19.

**Método:**

Estudio cualitativo descriptivo y exploratorio, guiado por el concepto de Calidad de Vida de la Organización Mundial de la Salud. Participaron 24 niños, con edades entre siete y nueve años, 11 meses y 29 días, diagnosticados con COVID-19. Los datos fueron recolectados a través de entrevistas semiestructuradas y sometidos a análisis temático deductivo.

**Resultados:**

Los niños entendían lo qué es la COVID-19, su alta letalidad y transmisibilidad y sus formas de contagio y prevención. La COVID-19 ha sido identificada como algo negativo. La experiencia de los niños enfermando por COVID-19 estuvo permeada por cambios en sus rutinas, especialmente por tener actividades limitadas al hogar, cambios emocionales y síntomas físicos por la infección con el virus.

**Conclusión:**

Los niños comprendieron la gravedad de la pandemia e identificaron los cambios que se habían producido. También expresaron comprensión sobre la enfermedad y su control. Conocer la experiencia de enfermedad de los niños puede orientar las acciones de cuidado dirigidas a ellos, reconociendo que la comprensión adecuada de lo que viven los niños puede contribuir al enfrentamiento de la enfermedad y su participación en las acciones de control.

## INTRODUCTION

*Coronavirus disease* 2019 (COVID-19), caused by the virus Severe Acute Respiratory Syndrome Coronavirus 2 (SARS-CoV-2), was responsible for a global health crisis, being considered the greatest global threat to public health at the time^(1)^. The high transmissibility of the virus did not differ within the adult and pediatric population. However, children were identified as a group showing fewer symptoms of COVID-19, although they were not immune to the illness and its serious forms^(2)^.

Information from the Brazilian Ministry of Health indicates that, from December 2019 to December 2021, of the 23,277 confirmed cases of COVID-19 in children aged zero to 11 years, 1,449 died^(3)^. Data collected by the United Nations Children’s Fund (UNICEF) in 91 countries show that, from the beginning of the pandemic until June 2022, COVID-19 was the main cause of death for 5,376 children under five years of age in the world, so that Brazil accounts for around one in five of these deaths^(4)^. From January to December 2022, Brazil recorded one death per day of children aged between six months and five years diagnosed with COVID-19. In total, there were 314 deaths in this age group in the period^(5)^.

To reduce the transmission of the virus, prevention measures were implemented, including social distancing. Although necessary, this measure reduces social cohesion and individuals’ access to public services and institutions that make up their social network. The closure of educational institutions, daycare centers, churches and the interruption of social protection services, are changes that contribute to the increase or worsening of situations of violence already established against women, children, and adolescents^(6)^ and to children’s mental health impairment^(7)^. The repercussions of social distancing on mental health are wide-ranging and can be long-lasting, with emotional and behavioral problems standing out in children^(8)^.

Children’s understanding of the situations they experience is different from that of adults, being influenced by the information they access. In this regard, in the process of becoming ill with COVID-19, children may have perspectives that limit their ability to deal with becoming ill with SARS-CoV-2, and may present better or worse responses to the situations they experience. These responses have the potential to have an impact on their quality of life (QOL), defined by the World Health Organization’s Quality of Life Group (WHOQOL) as the individual’s perception of their life, in relation to their cultural context and value systems, as well as in relation to their objectives, expectations, standards, and concerns^(9)^.

It is by considering the relevance of individual aspects in situations of illness that investigating children’s experiences of becoming ill with COVID-19, with them being the informants themselves, can contribute with evidence for actions to fight the late effects of the pandemic and future health emergencies. The concept of QoL, by having as a reference the experiences of individuals and their multidimensionality, can contribute to expanding knowledge on the subject and supporting the training and practice of professionals to care for children and their families in situations of illness and social distancing.

Therefore, for this investigation, the following research question was defined: What is the experience of children regarding their illness from COVID-19? To answer this question, the objective was to describe children’s experience of becoming ill with COVID-19.

## METHOD

### Design of Study

This study is an excerpt from a larger research, called “Repercussions of social distancing and illness due to COVID-19 on the quality of life of children aged 7 to 9 years”^(10)^. This is an investigation with a qualitative approach of a descriptive and exploratory nature, based on the World Health Organization (WHO) concept of QoL^(9)^. The preparation of this article met the recommendations of the Consolidated Qualitative Research Reporting Criteria (COREQ)^(11)^.

### Local

The study participants were located on a database made available by the Municipal Health Department of Belo Horizonte (SMSA-BH) with information on children diagnosed with COVID-19. Children residing in four regions of the municipality were selected, representing the diversity of the Health Vulnerability Indexes (HVI). The HVI is calculated using socioeconomic and environmental variables to identify the health vulnerability of a population and inequalities in the epidemiological profile of social groups^(12)^.

### Population

Children between the ages of seven and nine years, 11 months and 29 days who were diagnosed with infection with the new coronavirus in Belo Horizonte, Minas Gerais, participated.

### Selection Criteria

The inclusion criteria for the participants were: being aged between seven and nine years, 11 months and 29 days, living in one of the four selected regions, and having been diagnosed with COVID-19 confirmed with the laboratory test Reverse Transcription-Polymerase Chain Reaction(RT-PCR). The exclusion criteria were the occurrence of hospitalization due to COVID-19 and impaired communication and psychological and/or psychiatric changes that could hinder the comprehension of the questions to be answered. The failure of the telephone call and the lack of information that would allow contact with the children’s families were also considered.

### Sample Definition

The sample was defined by convenience. There were 2433 reports of children infected with SARS-CoV-2 between January 2020 and August 2021. Of these, 1012 were excluded because they were not eligible for the study and another 593 were excluded due to the lack of contact information, such as the child’s name, guardian, telephone number, or address. There remained 828 notifications of children, of which only 373 were eligible according to the study criteria. The first author of this study collected information from the notification form of eligible children, such as name, address, telephone number and date of notification, and made telephone contact with the children’s caregivers aiming at presenting the study and inviting them to participate. Of the 373 eligible children, the primary caregivers of 50 were contacted. This contact occurred randomly, through a draw carried out on the website *https://www.random.org/*. Of the 50 caregivers, 24 agreed to participate. No child, after the guardian’s acceptance, refused to participate in the research. The collection was interrupted when it was found that the data collected so far was sufficient to respond to the objective of the study and that the participants interviewed were diverse in terms of characterization and living conditions.

### Data Collection

Data collection took place between July 12th and December 15th, 2021. During this period, vaccination against COVID-19 began in Brazil, while new variants of SARS-CoV-2 were identified. Childhood vaccination was approved on December 16, 2021 for children aged five to 11^(13)^.

All contacts with families were carried out by the first author, who is a nurse, with master’s degree in nursing, and experience in caring for children and conducting interviews. The first contact was via telephone, to introduce themselves, explain the objective of the research, and invite them to participate. Upon acceptance, a day and time were scheduled for the child and guardian to be interviewed.

During the invitation to participate in the research and before the beginning of the interview, the primary caregivers and children were consulted about the possibility of the children being alone during the interview, with no objection from any of them. The interviews took place via video calls, with the children and the first author in their respective homes, respecting the social distancing required during the pandemic. A semi-structured script was used for the interview, with the aim of collecting information from children about the experience of becoming ill due to COVID-19. After each interview, the researcher made field notes with her impressions related to each child.

The initially constructed interview script was tested with two children chosen intentionally. The interviews were analyzed by the first and last author, who verified the need for adjustments in the elaboration of the questions to respond to the objective of the study and to ensure the children’s understanding. Both interviews were discarded. A new version of the interview guide was prepared with main questions and supporting questions. This version was tested with four other children, aged between seven years and six months and nine years and four months, who varied in aspects such as biological sex, race/color, presence of symptoms during infection, and educational institution. Again, the information from the interviews was analyzed by the first and last author of the study, checking its adequacy. The four children were included in the final sample. The interview consisted of the following main questions: Do you know what coronavirus is? What did you do when you got sick? Since the beginning of the pandemic, have you ever wanted to do something that you couldn’t do? To protect ourselves and other people we have to stay at home. How do you feel about having to do this? How did you feel when you got sick from the coronavirus? Have you ever felt afraid since you were sick?

The interview began with a conversation between the first author and the child about what she liked to do, what she had done in the last few days for fun. After this initial dialogue, the children seemed comfortable and at ease, and the interview trigger questions were introduced. To facilitate understanding, the researcher tried to use terms similar to those that the children themselves used.

### Data Analysis and Treatment

The interviews were audio recorded and lasted an average of 29 minutes. They were transcribed in full by the main author, with support from Inqscribe^®^ Software and subjected to deductive thematic analysis^(14)^, guided by the concept of QoL^(9)^. The MAXQDA© software, version 20.2.1, was used to data organization, codification, intercoder validity checking, and data exploration support.

Data analysis was performed following the recommendations of Clark and Braun^(14)^ for thematic analysis. Five codes were defined based on the concept of QOL^(9)^. The interviews were initially coded simultaneously and independently by the first and second authors of this study. After this process, an agreement index of *Kappa* 0.32 was observed, indicating the need for conceptual alignment, which was carried out together with the last author. A new round of coding was carried out by the first and second authors, reaching a Kappa index of 0.76 for intercoder agreement. After coding five interviews, the process was conducted only by the main author and, whenever a doubt was identified in the application of the code, this doubt was discussed with the last author for consensus. The codes used for this study and their respective themes are described in Chart 1.

**Chart 1:** Construction of the category and subcategories – Belo Horizonte, MG, Brazil, 2020.

Construction of themes	
**Main theme:** The experience of children when they become ill with COVID-19.	
**Initial codes**	**Themes**
Information about COVID-19 and the pandemic.	Children’s knowledge about COVID-19
Physical well-being; Psychological and spiritual well-being.	Physical Symptoms and Emotional Changes experienced by children
Social well-being; Repercussions on the environment.	Routine changes

### Ethical Aspects

During the development of this work, aspects of Resolutions 466/12 and 580/2018 of the National Health Council (CNS) were taken into account. The study was initiated after approval by the Research Ethics Committee (COEP) of the Federal University of Minas Gerais, on November 13, 2020, under opinion no. 4.397.579, and by SMSA-BH, on November 15, 2020, under opinion no. 4.466.214.

To ensure the dimensions of research with children, two documents were used: i) the Free and Informed Consent Form (FICF), a document in which the primary caregiver attested to their consent for their child to participate; ii) and the Free and Informed Assent Term (FIAF), an instrument used to ensure children’s autonomy when choosing whether to participate in research, produced in accessible language, so that children in the pre-literacy stage and non-literate ones could understand the research. Acceptance was made by audio recording. A copy of the FICF and FIAF was sent to the family electronically or by mail, depending on preference.

To ensure the anonymity of the participants, the interviews were anonymized by replacing the child’s name with “C”, followed by the order number of the interview (e.g.: C11). Names identifying institutions, locations, and other people were excluded to ensure confidentiality.

## RESULTS

### Characterization of Participating Children

Twenty-four children participated, the majority of whom were female (n=15). Half of the children was declared by their parents to be white, seven as mixed race, three as black, one as indigenous, and one as yellow. Of the children interviewed, seven had some type of comorbidity, the most common of which were asthma and bronchitis. Nine children experienced the death of close family members. The presence of physical symptoms due to COVID-19 infection was reported by 21 of the 24 participating children.

The median time elapsed between the date of notification of the child’s SARS-CoV-2 infection and the date of the interview was 140.5 days. In relation to the date of notification, the minimum age at the time was seven years, two months, and 20 days, and the maximum age was nine years, three months and 15 days. Regarding the age of the children at the time of the interview, the minimum age was seven years and eight months, and the maximum age was nine years, ten months and six days.

### The Experience of Children When They Become Ill With Covid-19

The children’s reports allowed identifying aspects of their experiences of becoming ill with COVID-19. The fragments of their speeches were grouped giving rise to three themes: *Children’s knowledge about COVID-19; Changes in children’s routine; Physical Symptoms and Emotional Changes experienced by children*.

### Children’s Knowledge About Covid-19

When asked if they knew what the coronavirus was, despite not differentiating the concept between the pandemic, COVID-19 or the virus, the answers from most children suggest that they had general information on the topic. They described the coronavirus as a *small animal* (C13 and C16), an *invisible ball* (C18). They recognized the transmissibility of the disease by stating that it is a *very contagious virus*, which *spread* (C18) and *dominated the world* (C9). They also reinforced that the coronavirus can harm people, since it *makes people sick* (C13 and C16), *feel unwell* (C18) and that *it can kill* (C5). The disease caused by the virus was described by children as *very strong* (C16), which leaves people *admitted to hospital and also in serious condition* (C18), which *is killing people all over the world* (C23). The pandemic is described as a *virus that has spread throughout the world* (C8) and that *there is no cure* (C8), explaining the magnitude of this event on the world stage.

When asked how they learned about the disease, the children stated that they learned through their family, newspapers and schools: *my grandmother keeps talking* (C5), *I learned all this from my mother and father* (C6), *I hear people talking* (C11), *I heard in the news* (C3), *my teacher gave an activity about this* (C10).

### Changes in Children’s Routine

During the specific period of illness, some children reported that they were isolated in their room, or in contact only with people in their own home (C1, C8, C9, C12, C17). Even without showing symptoms, C12 says that during the period of infection with the virus she also remained *isolated from people, with only the family at home, without going out* (C12).

When children lived, or had the possibility of being in farms and places with leisure spaces, even during the period of coronavirus infection, these children did not remain isolated and restricted to the home environment, and maintained the practice of physical activities, as is the case of C11 and C24.

The changes observed in the children’s lifestyle habits, determined by the virus infection, occurred due to the interruption of daily activities carried out with the family, the impossibility of meeting people and talking as before, as exemplified by C23’s statement that I *couldn’t meet people and spend more time talking to my family as before*. In addition to changes in contact with people, children report changes in their studies or in some daily habits, such as having *to sleep away from my aunt* (C8), *no one could come here to my house and when they did I had to stay in my room, with the door closed* (C9), and *I stopped studying for a bit* (C11).

In addition to isolation, hygiene measures such as washing hands (C6, C8 and C23), using hand sanitizer (C5 and C19), and wearing a mask (C3, C12 and C19) were intensified. Among these prevention measures, the use of a mask was approached by most children as something annoying. They understand the importance of wearing it, but they see this need as an obligation to which they have been conditioned, as seen in expressions such as *Now we have to wear a mask at home and everywhere* (C23). Furthermore, they demonstrated their discomfort with using the mask: *I suffocate in that mask. It’s horrible!* (C17).

C12 had to stay in his room during the infection period and says he did *absolutely nothing!* He was left *just playing on the phone*. The use of electronic devices appears frequently in children’s reports, having been incorporated to: *study online* (C2), *talk to my friend* (C6), *watch videos on YouTube, watch Tiktok, record video on Instagram* (C9), *and sometimes to play a game or watch cartoons* (C14).

For some children, such as C1 and C8, becoming ill with COVID-19 did not bring changes to their routine, as they were already practicing social isolation. They stated that they did *all the same* (C1), *I was doing everything I do, watching the computer, watching television* (C8).

The interviews showed that the children who were able to understand and assess the lethality of the coronavirus as the cause of a disease that *can kills* (C23) mentioned social distancing so as not to contaminate them as a change in their routine (C18, C21, C6 and C8). For children who understood the high transmissibility of the virus, stating that it is *contagious* (C24) and that it’s *everywhere* (C14), the main changes mentioned were the intensification of sanitary measures, such as *wash hands* (C5, C10), *use hand sanitizer* (C5 and C10), *mask* (C10 and C24), and *stay at home* (C10, C14 and C17).

On the other hand, some of the children, despite understanding the severity of the disease, reported that *nothing changed* (C1, C2, C3, C4 and C13) in their routines during the period of infection with the new coronavirus. However, when examining these children’s life context, it is observed that their parents were married, had completed higher education, and the family income was equal to or greater than 9.9 minimum wages, lived in their own home and had the possibility of leisure outdoors. These living conditions characterize a different social context from other children and the expression of an also different experience of illness. Additionally, two children in this group (C1 and C2) were asymptomatic, which may have contributed to the perception of no change in routine.

In addition to C1 and C2, C11 was also an asymptomatic child. However, she mentioned the need to be isolated in her room and the fact that she could not leave the house as a change in her routine. Unlike C1 and C2, this child experienced a family death due to COVID-19.

### Physical Symptoms and Emotional Changes Experienced by Children

In this article, physical symptoms refer to the physiological changes experienced by children during the period of illness from COVID-19. Emotional changes address the feelings that permeated this experience of becoming ill.

### Physical Symptoms

During the period of SARS-CoV-2 infection, some children presented symptoms common to COVID-19. They mentioned *headache every night* (C4), *dizziness, body pain, weakness* (C4), *didn’t want to eat, runny nose and fever* (C5), *couldn’t gaze anything, couldn’t hear anything too loud because the headache appeared* (C17), *and very sick* (C17). Change in taste was also reported, being described as follows: *It seems that the food had no salt* (C9), as a way of expressing the difference in the flavor of the food.

For C20, becoming ill with COVID-19 had an impact on his health condition over time. After the infection, the child began to present recurrent dermatitis and says that the *skin became very fragile* (C20), requiring medical monitoring.

### Emotional Changes

The emotional changes that were present when the children became ill were characterized by feelings of sadness, anger, fear, longing, and loneliness.

Sadness was common in children’s speeches throughout the pandemic. In C1’s speech, the child says he/she felt sad and justifies it by the impossibility of attending establishments and, mainly, the impediment of visiting his/her grandmother. Feelings of sadness were also mentioned by C2 and C4 with the closure of establishments, especially the school. Different sources of sadness were reported, such as having to change the routine and stay at home: *I was very sad because I couldn’t go out and I’m a person who really likes going out* (C10); *sad because I miss my family* (C13), for being alone: *I felt sad when I was there alone, wanting to play with my friend*. (C6). C5 reinforces the feeling of loneliness that exists during the period of illness: *We get stuck a lot, alone, we don’t talk to anyone. [...] Uh, because you can’t go out, you can’t talk to people, you can’t hug, be close*.

Anger was also an evident feeling in the children’s speeches. C23 says he/she *got angry because he/she wanted to go out and have fun*. C4 also mentioned that he felt *angry because couldn’t do anything at home*. C5 also mentioned feelings such as anger and sadness regarding the need for distancing, since she could not attend school: *I was very sad and very angry! When I wanted to go to school and couldn’t* (C5).

Fear, however, was in evidence during the specific period of virus infection, especially the fear of dying: *I was very afraid (...) Because I’m so afraid of dying* (C9); *I was scared and worried about catching the virus and dying*. (C24). Children also reported the fear of becoming infected again, as exemplified by C17: *Uh, the things I’m already afraid of, such as catching COVID-19 again and getting that horrible headache!* In addition to the fear of new infection, children are afraid of developing a severe form of the disease: *I’m sadder this way, because I’m afraid of getting it again... then getting something rarer, more serious* (C12), and with that, having to *be hospitalized, intubated* (C10). C11 was also afraid of being hospitalized, especially considering that he had lost his paternal grandmother and uncle due to COVID-19: *Most of my relatives were in the hospital, so I was very scared, because my grandmother and uncle caught it and died* (C11). C5, who also had the experience of losing family members, reported the fear of becoming infected again and of other family members dying, *I’m afraid of getting it again and of someone dying* (C5).

Children also reported fear of *passing on to other people* (C1), especially more fragile family members, as in the case of C3, who reports: *my grandmother had COVID-19 too, so I was afraid she would die*. C11 says he was *afraid of my mother catching it*. Fear of stigma due to the disease also emerged among children during the period of infection. C8 reported that he/she did not tell his/her friend that he/she had COVID-19 because he/she was *afraid to tell her and that she would never want to play again* (C8).

Of the nine children who experienced family death due to the coronavirus, seven reported the need to move away from family and friends as a change in their routine (C5, C9, C11, C12, C17, C18, C19). Furthermore, when asked about how they felt during the infection, eight reported that the main feeling was *fear* (C5, C9, C11,C12,C17, C18, C19 and C20) of becoming infected again and dying and of losing another family member to the disease. The word *fear* (C9 and C23) was used to express the understanding of the magnitude of the virus and its lethality potential. The feelings of *sadness* (C1,C2, C4 and C10) and *anger* (C4, C5 and C23), in their turn, were used when they talked about the need to stay at home and isolate themselves from people close to them.

**Figure 1:**
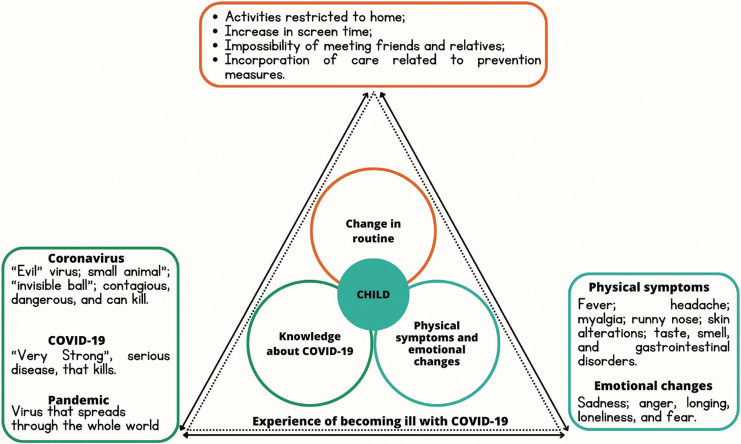
The perspective of children about their experience when they become ill with COVID-19. Source: Prepared by the authors (2022).

A schematic summary of the identified themes is presented in the Figure 1.

## DISCUSSION

This investigation confirmed the assumption that the experience of becoming ill with COVID-19 was influenced by the child’s understanding of this process and the meaning attributed to it. It is observed that the concrete situation of illness changed the routine of most children and demanded the intensification of measures to prevent and contain the transmission of the new coronavirus. Along with these changes, the child presented physical symptoms and emotional changes, which add to the illness process and/or the context in which the child is inserted. These dimensions discussed here and presented in the results coexist and interact, in a complex and dynamic process that is the process of becoming ill, especially in the context of COVID-19, marked by social distancing and the need to create a new way of living and interacting in the world and which have repercussions on QoL.

Through the children’s speeches, it was possible to know how they gave meaning to the information they received about the pandemic, based on their understanding of the world and their repertoire. They bring elements from their imagination to give materiality to their understanding, without minimizing the gravity and magnitude of real facts. Children, based on common images, use their experiences to translate the coronavirus. The description they have of their own illness caused by the virus is made using concrete information, apparently experienced by them. Therefore, it is clear that, even though children do not know how to express knowledge about the virus and its causes in the most scientific way, they do have information and understanding of some key elements surrounding the COVID-19 pandemic.

The knowledge that children have is acquired through close family members, or even from external environments, such as school and social media. A Brazilian investigation that interviewed 20 children aged eight and ten, showed that they directly associate the coronavirus with the disease it causes, to symptoms, transmissibility, and risk of death^(15)^, reinforcing the results found in this investigation.

Especially in the context of the pandemic, even with the large amount of information, a smaller portion was content produced in an accessible way for children. A survey developed from an online questionnaire, carried out with children aged seven to 12 years in the United Kingdom, Sweden, Brazil, Spain, Canada and Australia, showed that the children in the aforementioned study were aware that the coronavirus “spreads very quickly”, that “many people around the world are infected or have died”, that it “started in China”, is dangerous” and “can kill you”^(16)^. The ability of children to understand important elements about the outbreak of a contagious disease has already been demonstrated previously in relation to *Severe Acute Respiratory Syndrome* (SARS) in the 2003 epidemic^(17)^. In this study, for example, there is no misleading information in children’s speeches, even if we consider that most of the information conveyed about the coronavirus was aimed at adults.

The knowledge that children have about coronavirus is important as it has implications for the development of health-related behaviors(18). Through this study, it is clear how children understand that the virus can change their routine. The understanding that the virus is lethal and that it causes harm to people’s health, creates in children the need to adopt protective measures for themselves and those around them. During the period of illness, the children reported the need to remain isolated in their homes, maintaining contact only with family members living in the same household. In some cases, the children remained isolated in their rooms, with restricted access to other structures in the home. As a result, the possibilities for playing or going outdoors were further limited, and the time spent on electronic devices was greater. Using the device was an alternative found to bring the child closer to their relatives and friends, to fill their time in the face of social isolation, allow classes to continue and, to some extent, obtain information.

In this study, the children’s living conditions, especially housing with space for recreation, were decisive for their experience of social restriction. For these children, the changes were less significant, because they preserved the possibility of maintaining their activities outdoors and having space to play. In these life contexts, children had their right to play (Child and Adolescent Statute (ECA), art. 16, item IV) preserved, in a period in which it was neglected to ensure measures to reduce infections. Children in more vulnerable life contexts may have experienced illness in different ways than those presented in this study, as well as having a different perception of their QoL and the repercussions of their illness.

A study showed that social isolation and the consequent closure of schools, caused by the COVID-19 pandemic, affected children and their relationship with their families, significantly changing their routine, bringing new emotions, concerns and feelings, also changing partners, ways, spaces, and times to play. The longer stay at home, which does not always offer the best conditions for well-being, can lead to tensions and conflicts within the home and even episodes of violence against the child, which causes harm to their growth and development^(4,6)^. Furthermore, the increased use of screens during the pandemic may also pose immediate and long-term risks to child development^(4)^. Although technology is an important ally during the pandemic for maintaining children’s social and emotional ties, obtaining information through the use of screens can influence the responses expressed by them, since the dissemination of data on the numbers of infected, people who died due to the infection can generate emotional changes such as fear, stress, and anxiety^(19)^. Furthermore, exposure in virtual environments, without due supervision, favors the actions of sexual criminals and makes children vulnerable to this type of violence^(4)^.

In this investigation, although none of the children developed a severe form of the disease, for some of them the health conditions were more delicate, and a risk that exists in relation to COVID-19 is the persistence of symptoms even after infection. A case report about five Swedish children aged nine to 15 years, indicates that they had symptoms for six to eight months after their clinical diagnoses of COVID-19^(20)^. Among the participating children of this study, the case of C20 stands out, who, after the virus infection improved, continued to have skin lesions, requiring treatment and medical assistance.

The scenario of virus infection and numerous changes in children’s lives can generate feelings in them such as loneliness^(21)^, anxiety^(21–23)^, stress^(22)^, depressive symptoms^(21–24)^, anger^(24)^, fear^(21,24)^, and irritability^(25)^. A study carried out with children aged seven to 12 investigated the fear that children felt during the Swine Flu in 2009. The results indicated that children’s fear of swine flu was significantly related to their parents’ fear of this disease or direct experience with the disease^(25)^.

In this investigation, fear was related to the fact that little was known about the disease, fear of hospitalization, fear of death, fear of transmitting the disease, especially for those children who experienced cases of death of family members. Therefore, the approach to this topic with children must be done carefully, as the quality of communication with children about life-threatening illnesses and deaths has a long-term effect on their psychological well-being^(26)^.

By knowing the children’s perspectives on their experiences and, in particular, the changes experienced, whether physical or emotional, it is possible to focus on the work of nursing professionals to make them more effective, both in symptomatic control and in the emotional management of the child with COVID-19. Therefore, the study of the experiences lived by children in the context of illness due to COVID-19 and, in particular, knowing the symptoms and feelings experienced by them provides resources for improving the care provided by nursing professionals, whose practice focus is the care given to the human responses presented by people.

## CONCLUSION

The study findings show that the children had their own understanding of what the coronavirus is, its forms of contagion, illness, and its potential for lethality. Their feelings and the way they dealt with the pandemic seemed to be influenced by this knowledge.

The concrete situation of illness brought changes to the children’s routine. Daily activities were limited to the home, there was an increase in screen time, an intensification of hygiene and disease transmission control measures, and children could not see friends and family. Physical symptoms such as fever, headache, and runny nose were presented by the children, as well as emotional changes, including sadness, anger, loneliness, longing, and fear.

The limitation of this study was that it was carried out approximately a year and a half after the start of the pandemic. Thus, it was not possible to know the immediate repercussions of children who became infected at the beginning of the health emergency. Another limitation was the difficulty in contacting all children, which may suggest that the investigation did not reach children in vulnerable situations.

The need for systematic actions related to health education about the pandemic for children is identified, considering that this population was not targeted by the guidelines on coronavirus as they are not the main risk group. Moreover, further studies investigating the repercussions on the lives of these children in the long term are required, since the changes perceived may have the potential to last until adulthood.
